# Poly(I:C)-induced maternal immune activation causes elevated self-grooming in male rat offspring: Involvement of abnormal postpartum static nursing in dam

**DOI:** 10.3389/fcell.2023.1054381

**Published:** 2023-03-16

**Authors:** Xing-Yu Lan, You-Yu Gu, Ming-Juan Li, Tian-Jia Song, Fu-Jun Zhai, Yong Zhang, Jiang-Shan Zhan, Tobias M. Böckers, Xiao-Nan Yue, Jia-Nan Wang, Shuo Yuan, Meng-Ying Jin, Yu-Fei Xie, Wan-Wen Dang, Hai-Heng Hong, Zi-Rui Guo, Xue-Wei Wang, Rong Zhang

**Affiliations:** ^1^ Department of Neurobiology, School of Basic Medical Sciences, Peking University, Beijing, China; ^2^ Neuroscience Research Institute, Peking University, Beijing, China; ^3^ Key Laboratory for Neuroscience, Ministry of Education, National Health and Family Planning Commission, Peking University, Beijing, China; ^4^ Department of Integration of Chinese and Western Medicine, School of Basic Medical Sciences, Peking University, Beijing, China; ^5^ Institute for Anatomy and Cell Biology, Ulm University, Ulm, Germany; ^6^ Health Bureau of Kenli District, Dongying, China; ^7^ Autism Research Center, Peking University Health Science Center, Beijing, China

**Keywords:** poly (I:C), maternal immune activation, autism-like behavior, maternal behavior, dam, offspring

## Abstract

**Introduction:** Maternal immune activation (MIA) is closely related to the onset of autism-like behaviors in offspring, but the mechanism remains unclear. Maternal behaviors can influence offspring’s development and behaviors, as indicated in both human and animal studies. We hypothesized that abnormal maternal behaviors in MIA dams might be other factors leading to delayed development and abnormal behaviors in offspring.

**Methods:** To verify our hypothesis, we analyzed poly(I:C)-induced MIA dam’s postpartum maternal behavior and serum levels of several hormones related to maternal behavior. Pup’s developmental milestones and early social communication were recorded and evaluated in infancy. Other behavioral tests, including three-chamber test, self-grooming test, open field test, novel object recognition test, rotarod test and maximum grip test, were performed in adolescence of pups.

**Results:** Our results showed that MIA dams exhibit abnormal static nursing behavior but normal basic care and dynamic nursing behavior. The serum levels of testosterone and arginine vasopressin in MIA dams were significantly reduced compared with control dams. The developmental milestones, including pinna detachment, incisor eruption and eye opening, were significantly delayed in MIA offspring compared with control offspring, while the weight and early social communication showed no significant differences between the two groups. Behavioral tests performed in adolescence showed that only male MIA offspring display elevated self-grooming behaviors and reduced maximum grip.

**Discussion:** In conclusion, MIA dams display abnormal postpartum static nursing behavior concomitantly with reduced serum levels of testosterone and arginine vasopressin, possibly involving in the pathogenesis of delayed development and elevated self-grooming in male offspring. These findings hint that improving dam’s postpartum maternal behavior might be a potential regime to counteract delayed development and elevated self-grooming in male MIA offspring.

## Introduction

Autism spectrum disorder (ASD) is a heterogeneous group of refractory neurodevelopmental disability. It is characterized by social dysfunction as well as repetitive and stereotyped behavior (American Psychiatric Association, 2013). According to an umbrella review of evidence, up to 67 environmental risk factors and 52 biomarkers have been evaluated in the pathogenesis of ASD (Kim et al., 2019). However, the mechanism of ASD remains unclear with few effective therapies due to the high clinical and genetic heterogeneity (Bhat et al., 2014; Bhandari et al., 2020). It is of great significance to explore the mechanism of ASD, which might pave the road toward novel strategies in therapy.

The prevalence and disability-adjusted life years of ASD have increased rapidly in countries with high socio-demographic index from 1990 to 2019 (Solmi et al., 2022). According to the latest data, the global prevalence of autism is approximately 1/100 (Zeidan et al., 2022), while in China it is up to 0.70% (Zhou et al., 2020). Coincidentally, numerous epidemiologic studies provide evidence that maternal viral infection is growing rapidly in developed countries (Cannon and Davis, 2005; Ludwig and Hengel, 2009; Bate et al., 2010; Basha et al., 2014; Taniguchi et al., 2014) and has significant association with autism (Atladottir et al., 2010b). Croen et al. (2019) found that women who had an infection accompanied by a fever during the second trimester are more likely to have children with ASD. Several meta-analyses of clinical studies also indicate that maternal infection during pregnancy confers an increase in risk for autism in offspring (Jiang et al., 2016; Tioleco et al., 2021). Taken together, we speculate that maternal viral infection might be one of the most important factors contributing to the dramatic increase of ASD morbidity.

Maternal viral infection can induce maternal immune activation (MIA), which affects fetal neurodevelopment *via* cytokine storm (Estes and McAllister, 2016). These cytokines are closely related to the onset of ASD. For example, the level of MCP-1 is significantly elevated in the amniotic fluid of ASD individuals (Abdallah et al., 2012). MCP1 is secreted by PDGFRβ vascular wall cell. It is firstly activated in central nervous system (CNS) responding to inflammation and in turn increases neuronal excitability by promoting excitatory synaptic transmission in glutamatergic neurons of multiple brain regions (Duan et al., 2018). Elevated levels of IFN-γ, IL-4 and IL-5 in maternal serum are associated with increased risk for ASD in offspring (Goines et al., 2011). Aberrant level of IL-17 has been reported in several rodent models of ASD (Thawley et al., 2022). Maternal IL-17a could promote abnormal cortical development and autism-like behaviors in mice offspring (Choi et al., 2016). Poly(I:C)-induced MIA activates integrated stress response (ISR) in male but not female mice offspring *via* an IL-17a-dependent manner, which reduced global mRNA translation and altered nascent proteome synthesis (Kalish et al., 2021).

Rodent MIA offspring is likely to be predisposed to ASD. Lee et al. (2021) found that lipopolysaccharide (LPS)-induced MIA male offspring showed social behavior deficits, anxiety-like and repetitive behavior, hypomyelination and abnormal microbiota profile. *Mycobacterium* tuberculosis-induced MIA mice offspring displayed increased grooming behavior (Manjeese et al., 2021). In addition, Atanasova et al. (2023) reported that MIA exacerbated ASD related alterations in *Shank3*-deficient mice, suggesting the synergistic effects of MIA and genetic factors in the pathogenesis of ASD. However, the effects of anti-inflammatory or antioxidant on ASD are controversial (Hafizi et al., 2019; Pangrazzi et al., 2020). Although antibiotic used on pregnant women may modify the influence of MIA on increasing the risk of ASD in child (Holingue et al., 2020), the incidence of ASD would be reduced by only 12%–17% if maternal infections could be prevented or safely treated in a timely manner (Tioleco et al., 2021), suggesting that MIA induced ASD in offspring through other mechanisms.

Maternal parenting behavior plays an important role in prosocial behavior development of children (Tang et al., 2022). Abnormal maternal parenting behavior is common in parents of ASD children and closely linked to the severity of ASD (Maljaars et al., 2014). Recently, Zambon et al. (2022) found that MIA disrupts hypothalamic neurocircuits of maternal care behavior, hinting that disrupted maternal care behavior might be involved in the pathogenesis of ASD induced by MIA. Maternal behaviors are regulated by multiple hormones. Estradiol, progesterone and prolactin are main peripheral hormones playing a synergistic role in the regulation of maternal behavior (Keller et al., 2019). Many neurochemical molecules, such as oxytocin (OXT) and arginine vasopressin (AVP), are also involved in regulating maternal behavior. OXT can mediate the formation of mother-infant connection and enhance maternal care (Mitre et al., 2016), while the main function of AVP is regulating maternal aggression (Bayerl and Bosch, 2019). Mothers of autistic children showed lower plasma levels of OXT and AVP as well as a higher plasma level of testosterone (Xu et al., 2013). In addition, increased corticosterone level in mother decreases neural response to baby’s crying (Laurent et al., 2011). Kirsten et al. (2013) found that prenatal exposure to LPS increases maternal serum corticosterone level, causing placental injury and increasing IL-1β level in adult rat offspring, which are relevant to autism.

Polyinosinic-polycytidylic acid [poly(I:C)] is a synthetic nucleotide dimer which has a great effect on inducing interferon (Traynor et al., 2004). In medical research, poly(I:C) is often used to mimic a viral infection (Schwarze et al., 2016). Exposure to poly(I:C) during mid-pregnancy in rats can be used as a model investigating MIA (Vorhees et al., 2012; McColl and Piquette-Miller, 2021). Importantly, this kind of animal model has been regarded as a preclinical model for neurodevelopmental disorders, such as autism and schizophrenia (Haddad et al., 2020).

In this study, we evaluated postpartum maternal behavior as well as serum levels of sex hormones, OXT, AVP and corticosterone in poly(I:C)-induced MIA dams. Developmental milestones and behaviors of MIA offspring were also recorded and analyzed.

## Materials and methods

### Animals

Male and female Sprague-Dawley (SD) rats (270 g–350 g) were obtained from the Department of Experimental Animal Sciences, Peking University Health Science Center. Animals were housed individually with free access to food and water under a 12–12 h light-dark cycle. The humidity was 50% ± 10% and temperature was 23°C ± 2°C. This study was carried out following USA National Institutes of Health Guide for the Care and Use of Laboratory Animals. The protocols were approved by Peking University Animal Care and Use Committee (LA2020228).

### Construction of poly(I:C)-induced MIA rat model

We constructed the poly(I:C)-induced MIA rat model according to previous studies (Vorhees et al., 2012; McColl and Piquette-Miller, 2021). After acclimating for a week, 12-week-old female rats were paired with age-matched male rats. The day was considered embryonic day 0 (E 0) in the presence of a vaginal plug. Then pregnant rats were housed separately. On E 14, pregnant rats received a single intraperitoneal injection of either 8 mg/kg poly(I:C) (Sigma, P9582) or the same volume of 0.01M phosphate buffered saline (PBS). After weaning at postnatal day 21 (PND 21), offspring of the same group and sex but from different dams were randomly mixed with 4-5 per cage until the end of the experiments.

### Experimental groups

**FIGURE 1:**
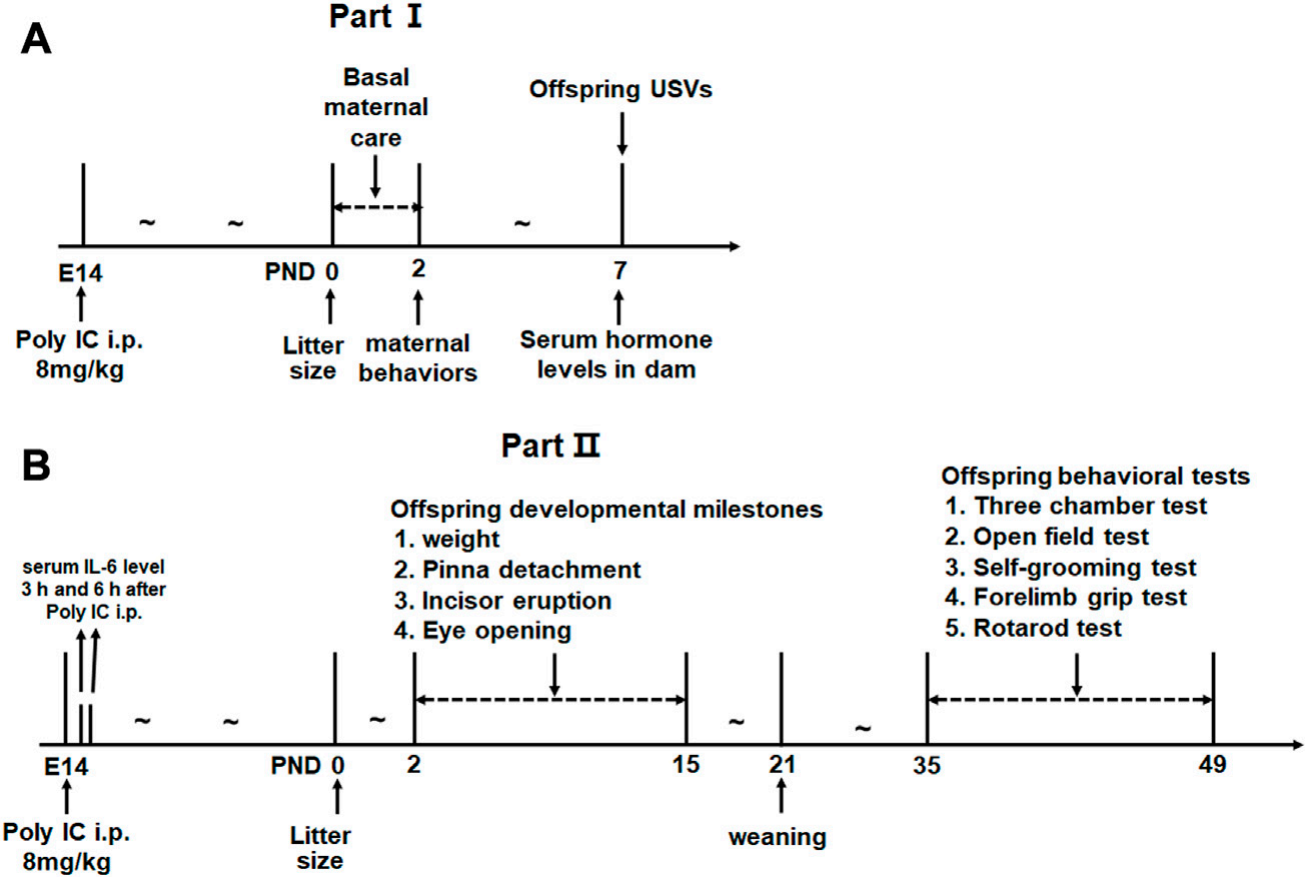
Experimental procedure. **(A)** Experimental design for Part I. **(B)** Experimental design for Part II.

The experiment was designed as 2 parts. In part I, 8 and 12 pregnant rats were subdivided into control group and MIA group respectively because poly(I:C) considerably increases the risk of resorption (Thaxton et al., 2013; Wang et al., 2015). We aimed to evaluate the postpartum maternal behavior and serum levels of several hormones related to maternal behavior in MIA dams. The litter size and the offspring’s early social communication were also evaluated in this part (see Figure 1A for experimental procedure). In part II, 5 and 3 pregnant rats were distributed into MIA group and control group, respectively. A total of 43 offspring (15 control males, 10 control females, 8 MIA males and 10 MIA females) were finally included in this part. We evaluated the litter size, developmental milestones and adolescent behavioral changes in MIA offspring. The serum IL-6 level of dams after poly(I:C) injection was also assessed in this part (see Figure 1B for experimental procedure).

### Serum IL-6 level of dams after poly(I:C) injection

The serum IL-6 level of dams were used to validate the establishment of MIA model (Parker-Athill and Tan, 2010). The blood of dams in part Ⅱ was collected *via* caudal vein 3 h and 6 h after poly(I:C) injection. Samples were placed at room temperature for 20 min and centrifuged at 1,600 g for 15 min to separate serum. The concentration of serum IL-6 was assessed following the kit instruction (CUSABIO, CSB-E04640r, Wuhan, China).

### Basal maternal care

The basal maternal care of dams was assessed as previously reported (Lonstein and Fleming, 2002). Cotton and tissue paper were given as nesting materials during the perinatal of female rats. The nests built by dams were scored by 2 experimenters who were blinded to the groups on PND 0, PND 1 and PND 2 (0 point: no nest, paper strips still scattered over entire floor of the cage; 1 point: poor nest, not all paper strips are used and the nest is flat; 2 point: fair nest, all paper is used but the nest is flat; 3 point: good nest, all paper is used and the nest wall is lower than 5 cm; 4 point: excellent nest, all paper is used and the nest wall is higher than 5 cm) and took the average. The nest score was the average of the 3-day scores. In addition, after delivery, the following indicators of offspring were recorded: with or without skin scar; attachment or removal of placenta; survival rate.

### Maternal behavior

Maternal nurturing behaviors of dams were assessed as previously reported (Lonstein and Fleming, 2002). On PND 2, dams were separated from the pups for 30 min and placed individually in the home cage. Pups were placed on a heating pad (37°C). After separation, dams were removed from the cage transitorily and pups were quickly placed in the four corners of the home cage. Then we placed dams back to the home cage and videotaped for 30 min. The maternal care nursing behaviors mainly include static nursing and dynamic nursing. Static nursing includes latency and duration of retrieval and crouching the pups. Dynamic nursing consists of the duration of hovering above and licking the pups. All the maternal behaviors were evaluated by an observer blinded to grouping. In addition, we analyze the dam’s grooming time and nest building time (Berger et al., 2018).

### Determination of serum testosterone, estradiol, progesterone, prolactin, corticosterone, oxytocin and AVP in dams

Serum levels of these hormones in dam were determined at PND 7. Blood was collected *via* decapitation and serum was separated. The levels of testosterone (CUSABIO, Wuhan, China), estradiol (CUSABIO, Wuhan, China), progesterone (CUSABIO, Wuhan, China), prolactin (CUSABIO, Wuhan, China), corticosterone (CUSABIO, Wuhan, China), oxytocin (Enzo life sciences, PA, USA) and AVP (Enzo life sciences, PA, USA) were assessed according to the kit instruction. All the 8 control dams in part II were included in this section. But among the 9 MIA dams, we failed to get blood in 1 MIA dam, and no enough serum was separated from another MIA dam. Finally, the serum hormone analytes had *n* = 8 per group except testosterone and corticosterone had *n* = 7 for the MIA group.

### Offspring’s developmental milestones

We recorded the offspring’s developmental milestones from PND 2 to PND 15, including body weight, pinna detachment day, incisor eruption day and eye-opening day. For each group, the number of pups achieving these developmental goals was daily recorded.

### Isolation-induced ultrasonic vocalizations (USVs)

Offspring’s early social communication was tested by isolation-induced USVs on PND 7 between 18:00 and 22:00 in a quiet environment with dim light. Offsprings were individually removed from the home cage and gently transferred to the test cage on a heating pad (37°C). USVs were recorded for 300 s for each pup and collected by an ultrasonic microphone (CM16/CMPA, Avisoft Bioacoustics, Berlin, Germany) hanging 25 cm above the cage floor. The connected amplifier (AUSG-116H, Avisoft Bioacoustics, Berlin, Germany) was set at a sampling frequency of 250 kHz with a 125 kHz low-pass filter. The recorded files were analyzed by Avisoft SASLab Pro (Version 4.52) using a fast Fourier transform (512 FFT-length, 100% frame size, hamming window, 50% time-window overlap). The number and duration of total USVs were recorded.

### Offspring’s behavioral tests in adolescence

Adolescent offspring began behavioral tests on PND 35. Male and female offspring were performed three-chamber test and self-grooming test on PND 35 and PND 37, respectively. In order to explore the underlying mechanism of elevated stereotypic behavior in male offspring, we performed open field test, novel object recognition test, rotarod test and maximum grip test on PND 39, PND 43, PND 46 and PND 49, respectively, in male offspring since rodent self-grooming has a strong association with sensation, motor and memory (Kalueff et al., 2016). All the behavioral tests were conducted in this particular order.

### Three-chamber test

Social preference and social novelty of offspring were evaluated on PND 35 by three-chamber test during the dark cycle (Dai et al., 2018). The three-chamber apparatus comprises three identical rectangular plexiglass chambers (40 cm × 34 cm × 24 cm). Each side chamber connected to the central chamber by a corridor. The subject rat was placed in the central chamber and explore freely for 5 min for habituation. Then two successive stages were followed. Stage I for social preference. An unfamiliar, weight and sex matched SD rat (Stranger 1) was locked in a wire cage and placed in one side chamber. An identical empty wire cage was placed in the other side chamber. The subject rat was placed in the central chamber and explored the three chambers for 10 min. Stage II for social novelty. Another unfamiliar, weight and sex matched SD rat (Stranger 2) was placed in the empty wire cage of the Stage I. The subject rat was then allowed to access to the three chambers freely for 10 min. During the experiment, the time spent in every chamber was automatically recorded. To minimize the impact from residual rat odors, the entire apparatus was thoroughly cleaned with 70% ethanol at the beginning of each trial.

### Self-grooming test

The self-grooming test performed on PND 37 is used to measure the severity of stereotyped behavior in offspring. The rat was placed into an empty cage similar to the home cage and explored freely for 10 min. Then rat behavior was video-taped for 10 min and total self-grooming time was calculated by a researcher who was blinded to grouping (Song et al., 2019). Self-grooming behaviors include: 1) wiping nose, face, head and ears with forepaws; 2) licking body, anogenital area and tail (Kalueff et al., 2016).

### Open field test

Anxiety behavior and spontaneous activity of male offspring were evaluated in open field test on PND 39. The subject rat was initially placed in the center of the acrylic box (100 cm × 100 cm × 40 cm) and explored freely for 10 min. Videos were processed by SMART software (v2.5.21, Panlab Harvard Apparatus). The total distances traveled in the open field represents spontaneous activity, while the time spent in the outer zone (the area ratio of center zone and outer zone is 1:3) represents anxiety behavior.

### Novel object recognition test

Learning and memory ability of male offspring were evaluated by the novel object recognition test during the dark period on PND 43 under dim red illumination according to a previous study (Song et al., 2019). A subject rat was placed in the arena (60 cm × 40 cm × 40 cm) for 10 min of habituation on the first and second day (PND 41 and PND 42). On the third day (PND 43), the rat was allowed to explore two identical objects in the arena for 20 min. One hour later, one of the two objects was replaced by a new object (with similar size but different color and shape). The rat was placed into the arena again exploring freely for 10 min and videotaped. The object exploration time was calculated by a researcher blinded to grouping. The object exploration behavior was defined as the nose of the rat touching the object or being oriented toward the object within 2 cm (Wang et al., 2011).

### Rotarod test

Male offspring’s motor ability was tested by a rotarod test. Subject rats were placed on the rotarod (Bioseb, France) for 5 min with a rotating speed of 4 rpm at the same time for 2 consecutive days (PND 44 and PND 45). At the same time of the third day (PND 46), subject rats were placed on the rotarod for 5 min with the rotating speed going from 4 rpm to 40 rpm. Every subject rat went through three repeated trials with an interval of 10 min. The latency to fall was recorded every time and the mean of three recordings was calculated.

### Maximum grip test

The male offspring’s maximum grip strength was measured by maximum grip test. All the male pups were trained to be adapted to the maximum grip test 2 consecutive days (PND 47 and PND 48). We gently placed the pups on the grid of the dynamometer (Bioseb, France) and pulled their tails in the opposite direction. On PND 49, the maximum grip strength exerted by the pup before losing grip was recorded. We repeated 3 measurements on each pup, allowing a 30 s recovery time between each measurement. The mean of 3 measurements on each pup was calculated.

### Statistical analysis

Data are presented as the mean ± SEM. Statistical analysis was conducted by unpaired t-test (quantitative data with normal distribution and equal variance) or Mann-Whitney U test (quantitative data with non-normal distribution). Shapiro-Wilk test was used to check normal distribution. Two-way ANOVA was used to analyze the pup weight. A paired t-test was used to determine within-group side preference in three-chamber test and object exploration time in novel object recognition test. Data were analyzed and graphed by GraphPad Prism software (version 8.0.1). A double-tailed *p* < 0.05 was considered statistically significant.

## Results

### Poly(I:C)-induced MIA dams showed acute elevated serum IL-6 level as well as reduced litter size

**FIGURE 2:**
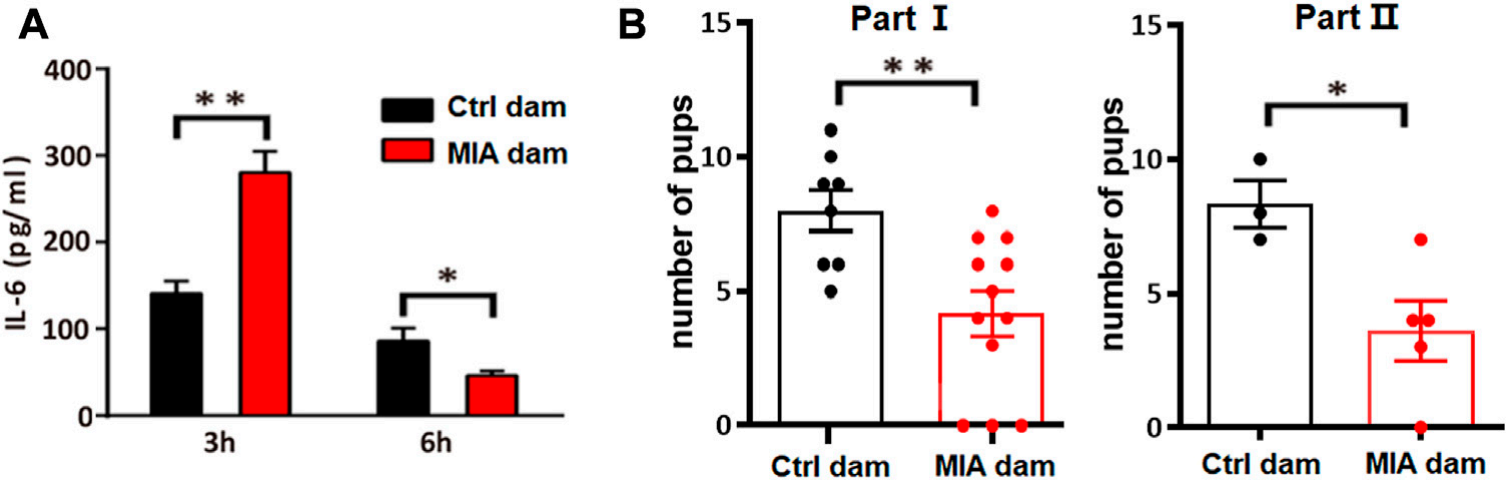
Model validation. **(A)** Serum level of IL-6 in dams 3 h or 6 h after Poly(I:C) injection. Ctrl dam, *n* = 3; MIA dam, *n* = 5; *p < 0.05 based on Unpaired t-test. **p < 0.01 based on Unpaired t-test. **(B)** Litter size of dams. Part I Ctrl dam, *n* = 8; MIA dam, n = 12; **p < 0.01 based on Unpaired t-test. Part II Ctrl dams, n = 3; MIA dams, *n* = 5; *p < 0.05 based on Unpaired t-test. Ctrl, control. MIA, maternal immune activation. Error bars in this figure represent mean ± SEM of the mean values of each experiment.

To validate the establishment of MIA model, we accessed the serum level of IL-6 in MIA dams 3 h and 6 h after poly(I:C) injection. The serum IL-6 level in MIA dam was significantly increased 3 h after poly(I:C) injection (Unpaired t-test, t = 4.059, df = 6, *p* = 0.0067, Figure 2A) but significantly decreased 6 h after poly(I:C) injection (Unpaired t-test, t = 3.032, df = 6, *p* = 0.0230, Figure 2A). In addition, we accessed the litter size of dams. The number of pups in MIA dams is significantly lower than that in control dams (Unpaired t-test, t = 3.204, df = 18, *p* = 0.0049, Figure 2B, part I; Unpaired t-test, t = 2.905, df = 6, *p* = 0.0272, Figure 2B, part II), which is consistent to previous studies (Kowash et al., 2019). This result indicated that poly(I:C) injection induced acute inflammation and reduced litter size in dams, suggesting that the MIA model construction is successful.

### Poly(I:C)-induced MIA dams showed abnormal static nursing behavior but normal basic care and normal dynamic nursing behavior

**FIGURE 3:**
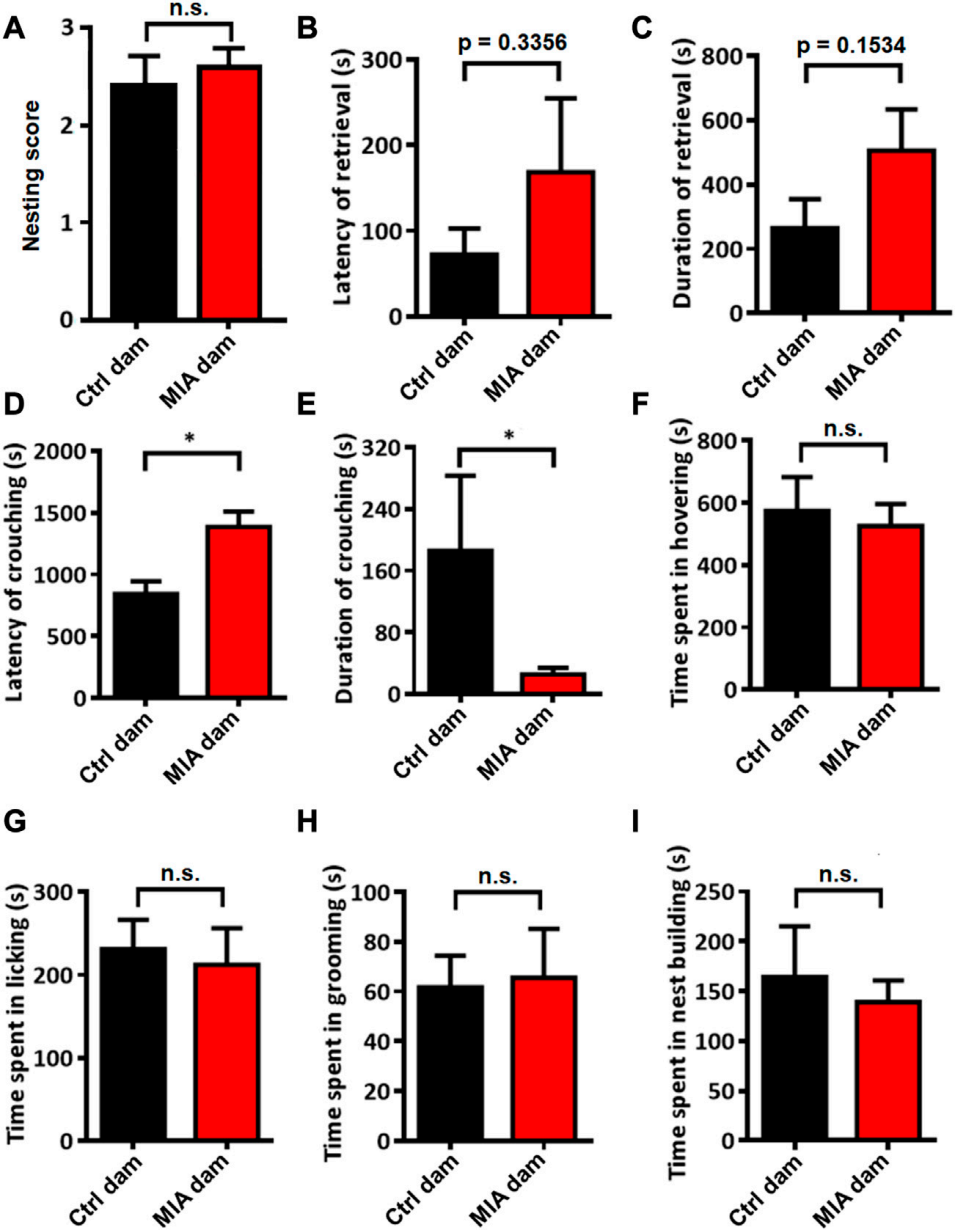
Postpartum maternal behavior in Ctrl and MIA dams. **(A)** Nesting score. Ctrl dam, *n* = 8; MIA dam, *n* = 9. Mann-Whitney U test. **(B–E)** Static nursing. Ctrl dam, *n* = 8; MIA dam, *n* = 9. **(B)** Latency of retrieval. *p* = 0.3356 based on Mann-Whitney U test. **(C)** Duration of retrieval. *p* = 0.1534 based on Unpaired t-test. **(D)** Latency of crouching. *p < 0.05 based on Mann-Whitney U test. **(E)** Duration of crouching. *p < 0.05 based on Mann-Whitney U test. **(F–G)** Dynamic nursing. Ctrl dam, *n* = 8; MIA dam, *n* = 9. **(F)** Time spent in hovering. Unpaired t-test. **(G)** Time spent in licking. Unpaired t-test. **(H)** Time spent in grooming. Ctrl dam, *n* = 8; MIA dam, *n* = 9. Unpaired t-test. **(I)** Time spent in nest building. Ctrl dam, *n* = 8; MIA dam, *n* = 9. Mann-Whitney U test. Ctrl, control. MIA, maternal immune activation. Error bars in this figure represent mean ± SEM of the mean values of each experiment.

We evaluated the postpartum maternal behavior of dams. The nesting score between the 2 groups showed no significant difference (Mann-Whitney U test, U = 33.5, *p* = 0.7529, Figure 3A). The placental adnexa tissue of all pups was cleaned and all the pups survived with no skin lesions (data not shown). These results suggested that the basal maternal care of MIA dams did not change compared with control dams. Regarding maternal behavior, the latency (Mann-Whitney U test, U = 25.5, *p* = 0.3356, Figure 3B) and duration (Unpaired t-test, t = 1.504, df = 15, *p* = 0.1534, Figure 3C) of retrieving the pups tended to be elevated in MIA group, while the latency of crouching the pups is significantly elevated (Mann-Whitney U test, U = 11, *p* = 0.0183, Figure 3D) and the duration of crouching the pups is significantly decreased in MIA group (Mann-Whitney U test, U = 15, *p* = 0.0480, Figure 3E). These results indicate that static nursing in MIA group is abnormal. The time spent in hovering above (Unpaired t-test, t = 0.3803, df = 15, *p* = 0.7091, Figure 3F) and licking (Unpaired t-test, t = 0.3257, df = 15, *p* = 0.7492, Figure 3G) the pups also showed no significant differences between control and MIA group, suggesting that the dynamic nursing did not change in MIA dams. We also evaluated the time spent in grooming (Unpaired t-test, t = 0.1559, df = 15, *p* = 0.8782, Figure 3H) and nest building (Mann-Whitney U test, U = 33, *p* = 0.8148, Figure 3I), which showed no significant differences between 2 groups.

### Poly(I:C)-induced MIA dams showed reduced serum levels of testosterone and AVP

**FIGURE 4:**
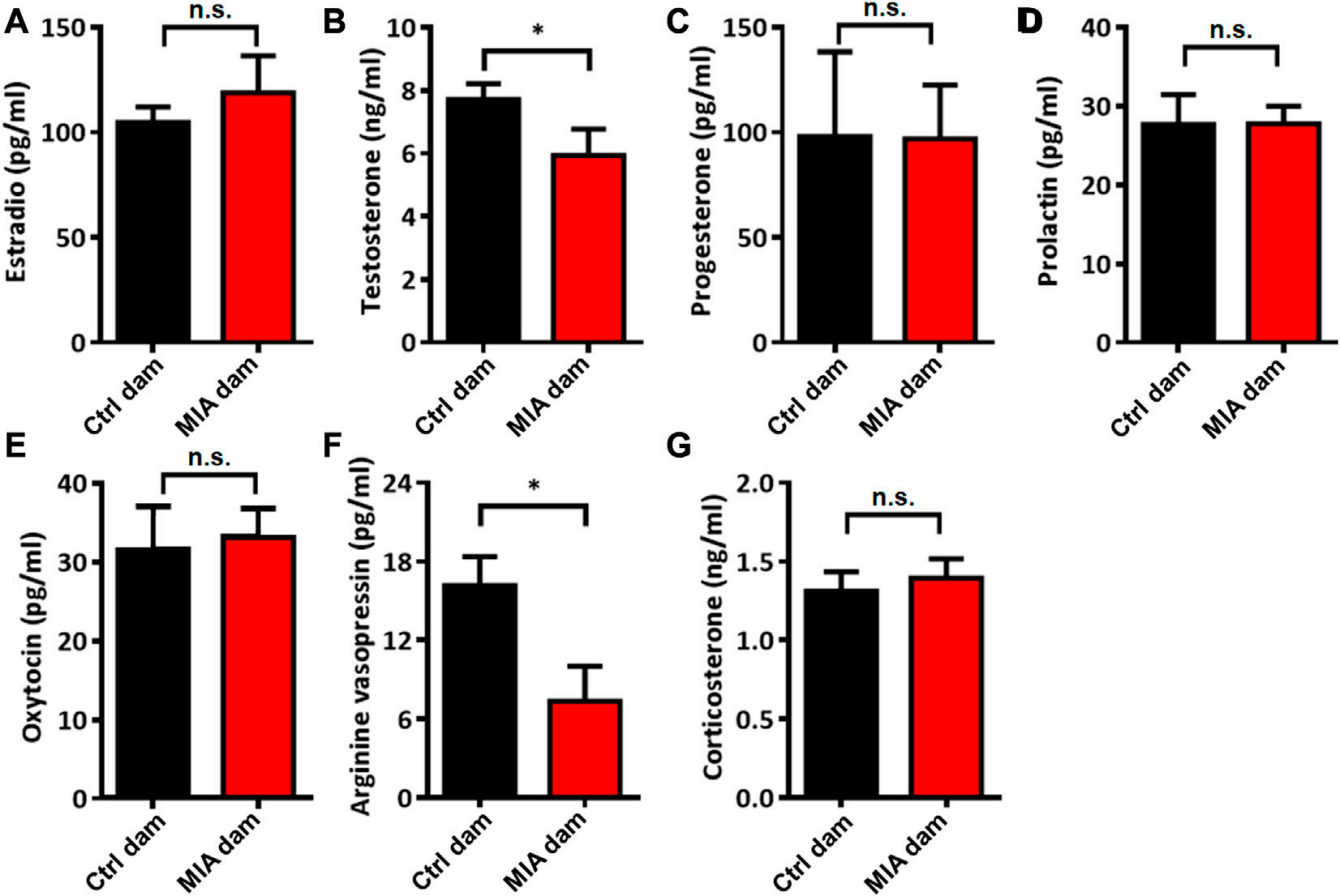
Serum levels of several hormones in dams on PND 7. **(A)** Estradio. Ctrl dam, *n* = 8; MIA dam, *n* = 8. Unpaired t-test. **(B)** Testosterone. Ctrl dam, *n* = 8; MIA dam, *n* = 7. *p < 0.05 based on Mann-Whitney U test **(C)** Progesterone. Ctrl dam, *n* = 8; MIA dam, *n* = 8. Unpaired t-test. **(D)** Prolaction. Ctrl dam, *n* = 8; MIA dam, *n* = 8. Unpaired t-test. **(E)** Oxytocin. Ctrl dam, n = 8; MIA dam, n = 8. Unpaired t-test. **(F)** Arginine vasopressin. Ctrl dam, *n* = 8; MIA dam, *n* = 8. *p < 0.05 based on Unpaired t-test. **(G)** Corticosterone. Ctrl dam, *n* = 8; MIA dam, *n* = 7. Unpaired t-test. Ctrl, control. MIA, maternal immune activation. Error bars in this figure represent mean ± SEM of the mean values of each experiment.

To evaluate the potential mechanisms of abnormal static nursing behavior in poly(I:C)-induced MIA dams, we evaluated the serum levels of several hormones related to maternal behavior. We found that the serum levels of testosterone (Mann-Whitney U test, U = 8, *p* = 0.0205, Figure 4B) and AVP (Unpaired t-test, t = 2.548, df = 14, *p* = 0.0232, Figure 4F) in MIA dams were significantly reduced compared with control dams. The serum levels of estradio (Unpaired t-test, t = 0.7375, df = 14, *p* = 0.4730, Figure 4A), progesterone (Unpaired t-test, t = 0.02518, df = 14, *p* = 0.9803, Figure 4C), prolactin (Unpaired t-test, t = 0.01691, df = 14, *p* = 0.9867, Figure 4D), oxytocin (Unpaired t-test, t = 0.2538, df = 14, *p* = 0.8033, Figure 4E) and corticosterone (Unpaired t-test, t = 0.4756, df = 13, *p* = 0.6422, Figure 4G) showed no significant differences between the 2 groups.

### Poly(I:C)-induced MIA offspring showed delayed developmental milestones with normal weight and normal early communication

**FIGURE 5:**
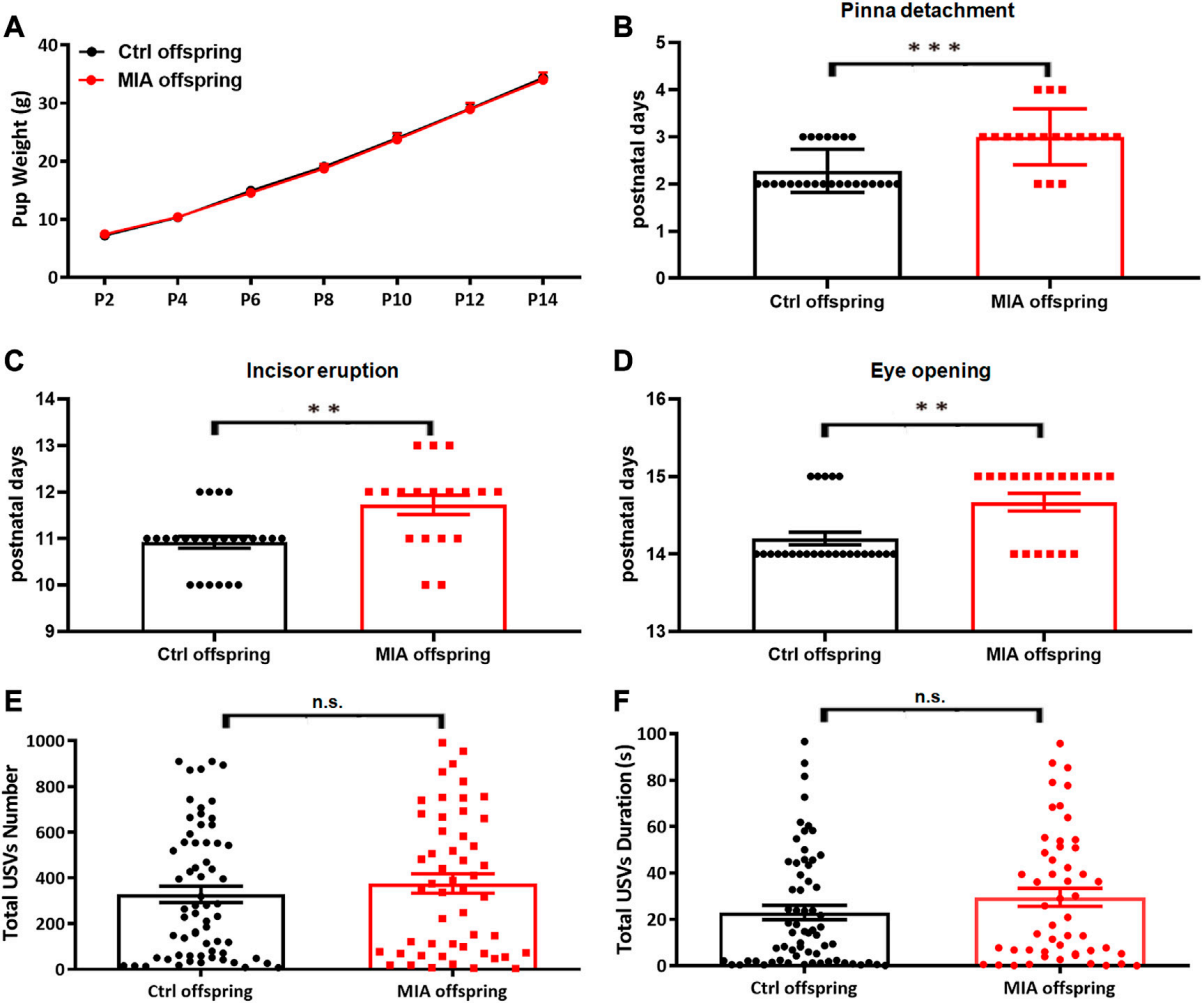
Offspring’s developmental milestones and early social communication. **(A–D)** Offspring’s developmental milestones. Ctrl offspring, *n* = 25; MIA offspring, *n* = 18. **(A)** Weight. Two-way ANOVA. **(B)** Pinna detachment. ***p < 0.001 based on Mann-Whitney U test. **(C)** Incisor eruption. **p < 0.01 based on Mann-Whitney U test. **(D)** Eye opening. **p < 0.01 based on Mann-Whitney U test. **(E,F)** Isolation-Induced USVs in offspring. Ctrl offspring, *n* = 64; MIA offspring, *n* = 51. **(E)** Total USVs number. Mann-Whitney U test. **(F)** Total USVs duration. Mann-Whitney U test. Ctrl, control. MIA, maternal immune activation. Error bars in this figure represent mean ± SEM of the mean values of each experiment.

To evaluate the developmental milestones in poly(I:C)-induced MIA offspring, we recorded the pup’s weight, pinna detachment, incisor eruption and eye opening from PND 2 to PND 15. The pup’s weight showed no significant difference between the two groups (two-way ANOVA, df = 6, *p* = 0.9466, Figure 5A), while the day of pinna detachment (Mann-Whitney U test, U = 90, *p* < 0.001, Figure 5B), incisor eruption (Mann-Whitney U test, U = 108, *p* = 0.0018, Figure 5C) and eye opening (Mann-Whitney U test, U = 120, *p* = 0.0039, Figure 5D) in MIA offspring were significantly later than that in control offspring, suggesting that the developmental milestones were significantly delayed in MIA offspring. The early social communication in offspring was evaluated on PND7. The isolation-induced USVs showed that total USVs number (Mann-Whitney U test, U = 1490, *p* = 0.4257, Figure 5E) and total USVs duration (Mann-Whitney U test, U = 1431, *p* = 0.2601, Figure 5F) were comparable between MIA and control group, implying that MIA offspring had normal early social communication.

### Male MIA offspring showed elevated self-grooming behaviors and reduced maximum grip

**FIGURE 6:**
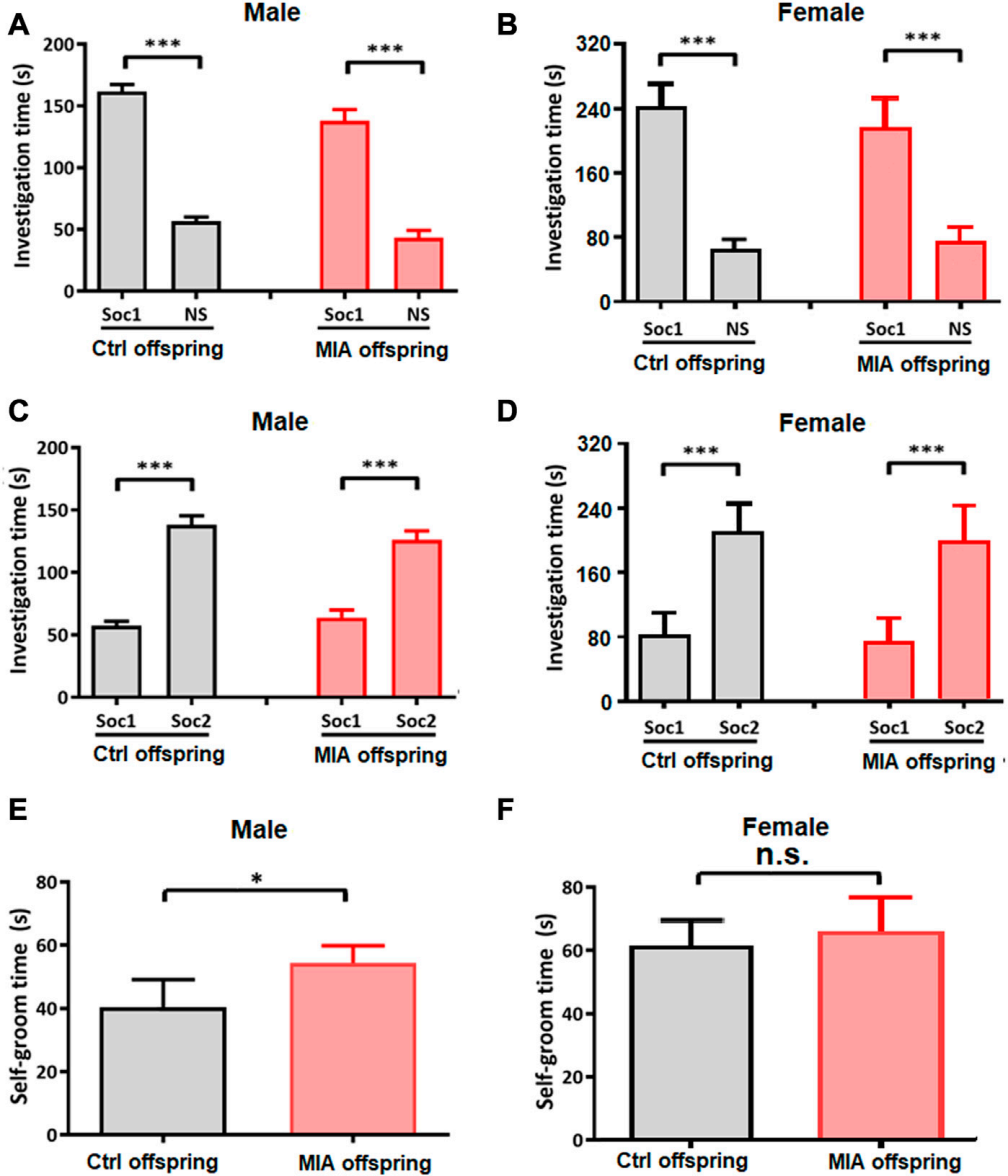
Offspring’s autism-like behaviors. **(A–D)** Three-chamber test. Ctrl males n = 15; Ctrl females *n* = 10; MIA males *n* = 8; MIA females n = 10. **(A)** Social preference of male offspring. ***p < 0.001 based on paired t-test. **(B)** Social preference of female offspring. ***p < 0.001 based on paired t-test. **(C)** Social novelty of male offspring. ***p < 0.001 based on paired t-test. **(D)** Social novelty of female offspring. ***p < 0.001 based on paired t-test. **(E,F)** Self-grooming test. Ctrl males *n* = 15; Ctrl females *n* = 10; MIA males *n* = 8; MIA females *n* = 10. **(E)** Self-grooming time of male offspring. *p < 0.05 based on Unpaired t-test. **(F)** Self-grooming time of female offspring. Unpaired t-test. Ctrl, control. MIA, maternal immune activation. Error bars in this figure represent mean ± SEM of the mean values of each experiment.

In order to assess the autism-like behaviors in MIA offspring, we performed three-chamber test and self-grooming test on PND 35 and PND 37, respectively. In three-chamber test, at Stage I (social preference), both control and MIA offspring spent more time in the side with Stranger 1 than in the empty cage (paired t-test; control male, t = 18.73, df = 14, *p* < 0.001; control female, t = 15.25, df = 9, *p* < 0.001; MIA male, t = 10.10, df = 7, *p* < 0.001; MIA female, t = 9.358, df = 9, *p* < 0.001; Figures 6A, B), while at Stage II (social novelty), both control and MIA offspring spent more time in proximity to Stranger 2 than Stranger 1 (paired t-test; control male, t = 10.11, df = 14, *p* < 0.001; control female, t = 6.698, df = 9, *p* < 0.001; MIA male, t = 5.999, df = 7, *p* < 0.001; MIA female, t = 6.097, df = 9, *p* < 0.001; Figures 6C, D), showing that both male and female MIA offspring had normal social preference and social novelty. Self-grooming test showed that the self-grooming time was significantly elevated in male MIA offspring (Unpaired t-test, t = 2.581, df = 21, *p* = 0.0174, Figure 6E) but not changed in female MIA offspring (Unpaired t-test, t = 0.5895, df = 18, *p* = 0.5629, Figure 6F).

**FIGURE 7:**
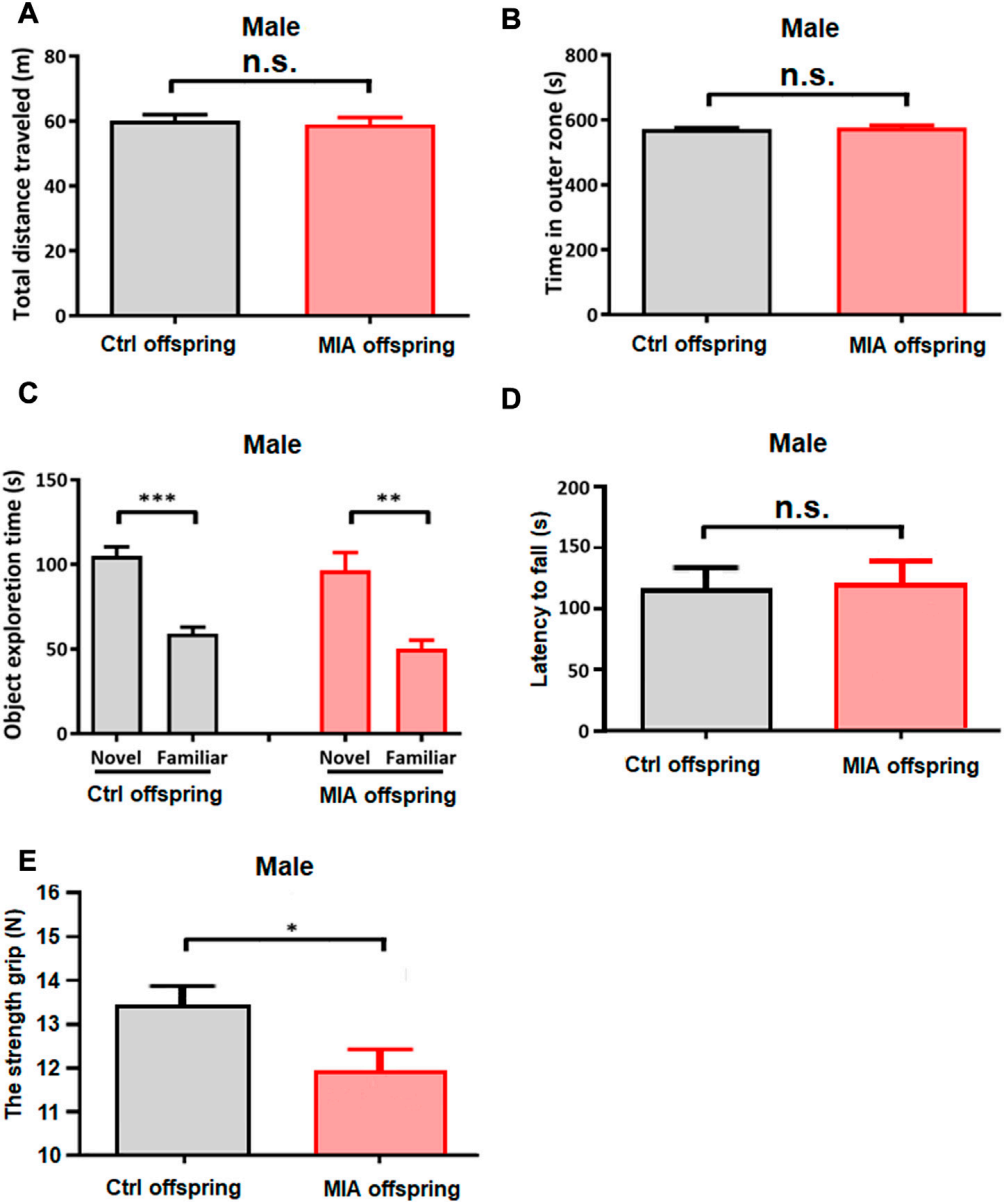
Male offspring’s other behavioral tests. **(A,B)** Open field test of male offspring. Ctrl males *n* = 15; MIA males *n* = 8. **(A)** Total distance traveled. Unpaired t-test. **(B)** Time spent in outer zone. Unpaired t-test. **(C)** Novel object recognition test of male offspring. Ctrl males *n* = 15; MIA males n = 8. **p < 0.01 based on paired t-test. ***p < 0.001 based on paired t-test. **(D)** Rotarod test of male offspring. Ctrl males *n* = 15; MIA males n = 8. Unpaired t-test. **(E)** Maximum grip test of male offspring. Ctrl males *n* = 13; MIA males *n* = 7. *p < 0.05 based on Mann-Whitney U test. Ctrl, control. MIA, maternal immune activation. Error bars in this figure represent mean ± SEM of the mean values of each experiment.

In order to explore the motor ability potentially related to elevated self-grooming behavior in male MIA offspring, open field test, novel object recognition test, rotarod test and maximum grip test were performed on PND 39, PND 43, PND 46 and PND 49, respectively. In open field test, MIA and control male offspring travelled comparable distance in the experimental arena (Unpaired t-test, t = 0.3804, df = 21, *p* = 0.7069, Figure 7A) and spent comparable time in the outer zone (Unpaired t-test, t = 0.6431, df = 21, *p* = 0.5260, Figure 7B), hinting that the anxiety behavior and spontaneous activity did not change in male MIA offspring. In novel object recognition test, both control and MIA male offspring spent more time exploring novel object than familiar object (paired t-test, Ctrl male, t = 7.326, df = 14, *p* < 0.001, MIA male, t = 4.391, df = 7, *p* = 0.0023, Figure 7C), showing that male MIA offspring had normal learning and memory ability. Rotarod test showed that the latency to fall from the rotarod showed no significant differences between control and MIA offspring (Unpaired t-test, t = 0.2923, df = 21, *p* = 0.7744, Figure 7D), suggesting that male offspring’s motor ability did not differ between the 2 groups. Maximum grip test showed that the maximum grip of male MIA offspring was significantly reduced (Mann-Whitney U test, U = 8, *p* = 0.0205, Figure 7E), implying that the muscle of male MIA offspring was weak.

## Discussion

In the current study, we established a poly(I:C)-induced MIA rat model to evaluate postpartum maternal behavior in MIA dams and autism-like behaviors in MIA offspring. MIA dams showed reduced litter size and abnormal static nursing behavior. The serum levels of testosterone and AVP significantly decreased in MIA dams. The early developmental milestones in MIA offspring were significantly delayed with no significant changes in weight and social communications. Both male and female MIA offspring have normal social preference and social novelty, while self-grooming behaviors are significantly elevated in male but not female MIA offspring. The strength grip of male MIA offspring was significantly reduced. Open field test and novel object recognition test showed no significant differences between the two groups. Our results hinted that abnormal maternal behavior in MIA dams might play an important role in the pathogenesis of delayed development and elevated self-grooming behaviors in male rat offspring.

MIA has been linked to an increased risk of ASD in offspring through affecting the development of the CNS *via* inflammatory molecules (Ciaranello and Ciaranello, 1995; Atladottir et al., 2010a). A recent study reported that MIA destroys hypothalamic neurocircuits of maternal care behavior (Zambon et al., 2022), which brings us a new clue that abnormal maternal behavior might be involved in the pathogenesis of autism-like behavior in MIA offspring. We found that MIA dams showed elevated latency and decreased duration of crouching the pups. The time spent in hovering above and licking pups as well as grooming and nest building time did not change between the 2 groups. These results are not consistent with a previous study reporting that poly(I:C)-induced MIA C3H/He mice dams showed reduced licking/grooming behaviors and elevated nesting building time (Berger et al., 2018). We speculate that behavioral and immunological effects of MIA on dams are strain-dependent. In addition, the litter size of MIA dams was significantly reduced compared with that of control dams. A previous study reported that reduction of litter size increased arched-back posture and licking pups in lactating rats (Enes-Marques and Giusti-Paiva, 2018), implying that litter size reduction might affect the results of postpartum maternal behavior. The influence of litter size should be eliminated in our future studies to make our conclusions more rigorous.

Furthermore, we evaluated serum levels of several hormones related to maternal behavior. We collected the serum of dams on PND 7 after pups’ early social communication was detected. Results showed that the serum levels of testosterone and AVP were significantly reduced in MIA dams. AVP has the twin peptide with OXT and plays a similar role in maternal behavior regulation (Bosch and Neumann, 2012) and aggression (Bosch and Neumann, 2012; Carter, 2017). Our previous clinical study reported that the plasma AVP level in mothers of ASD children tended to be lower than that of normal children (Zhang et al., 2016). It is interesting that the serum level of testosterone is significantly decreased in MIA dams, which is inconsistent with previous clinical findings, reporting that the serum level of testosterone is significantly elevated in mothers of ASD individuals (Ruta et al., 2011; Palomba et al., 2012; Xu et al., 2013). For there is still no direct evidence from the women experiencing inflammation, the reason of decrease of testosterone in MIA model needs more in-depth researches.

Self-grooming is a complex innate behavior frequently performed in rodents with an evolutionary conserved sequencing pattern. It is related with not only pattern of action but multiple motor abilities. Self-grooming behavior is regulated by multiple brain regions, including hypothalamus, striatum, neocortex, amygdala, brainstem and cerebellum (Kalueff et al., 2016). Elevated self-grooming behavior has been reported in many neurological and neuropsychiatric disorders, such as schizophrenia and ASD (American Psychiatric Association, 2013). In this study, we found that self-grooming behaviors were significantly elevated in male MIA offspring but not changed in females, indicating that MIA-induced autism-like behavior showed significant sex differences. The maximum grip of male MIA offspring was significantly reduced, implying that the muscle of male MIA offspring was weak. Haida *et al.* reported that male MIA mice offspring show reduced motor development and coordination deficits, as well as a significant decrease in the number of Purkinje cells in cerebellum and neurons in the motor cortex (Haida et al., 2019). In addition, reduced litter size can increase repetitive and stereotyped movements in offspring (de Novais et al., 2021). Similar results have also been reported in MIA mice offspring, showing that the autism-like behavior is more serious in male MIA offspring than that in females (Carlezon et al., 2019; Haida et al., 2019). It is well-acknowledged that the incidence of ASD is obviously male biased (Maenner et al., 2021). But the male-to-female ratio of ASD is heterogeneous, 6-16:1 in mild ASD population and 1-2:1 in severe ASD population (Fombonne, 2009; Jacquemont et al., 2014), suggesting that pregnancy infection is likely to cause mild ASD in offspring. Carlezon *et al.* reported in MIA offspring that the anti-inflammatory factors are decreased in males and increased in females (Carlezon et al., 2019), which to some extent explained the male biased morbidity of autism-like behavior induced by MIA. Taken together, we speculate that elevated self-grooming behavior in male MIA rat offspring is caused by multiple factors, including abnormal maternal behaviors, reduced litter size as well as immunological factors. The long-term effects of the maternal gestational environment or maternal behavior on pup phenotype need to be further evaluated.

However, results from different studies are heterogeneous. For example, Malkova et al. (2012) found that poly(I:C)-induced MIA mice offspring displayed reduced early social communication, decreased sociability and increased repetitive/stereotyped behaviors. Lee et al. (2021) constructed the MIA rat model by intraperitoneally injecting LPS on pregnant rats, finding that LPS-induced MIA rat offspring show reduced social ability and increased anxiety-like and repetitive behavior. Gzielo et al. (2021) constructed MIA rat model by intraperitoneal injection of 5 mg/kg poly(I:C) on PND 15, finding that male offspring showed deficits in social play behaviors, while elevated repetitive behaviors were found in both sexes. These phenomena are also appearing in the real world. During the COVID-19 pandemic, the potential effects of pregnancy SARS-CoV-2 infection on maternal and perinatal outcomes are controversial (Vergara-Merino et al., 2021). The outcomes of pregnancy and newborn can be affected by the symptoms and severity of COVID-19 as well as the infection time. Comparing with asymptomatic patients, symptomatic pregnant woman infected with SARS-CoV-2 are more likely to suffer from cesarean (Jenabi et al., 2022) or premature birth (Vimercati et al., 2022; Wilkinson et al., 2022). Fetuses born to symptomatic COVID-19 pregnant women have a significantly higher risk of suffering from low body weight comparing with those born to asymptomatic pregnant women (Jenabi et al., 2022). No significant relationship was found between asymptomatic pregnant women and fetal growth restriction (Narang et al., 2023). Fetuses born to SARS-CoV-2-infected women are more likely to suffer from neuromotor developmental disorders (Fajardo Martinez et al., 2023). They also had lower scores in communication, problem solving and personal-social domains (Cheng et al., 2021). Ayed et al. (2022) found that pregnant women infected by SARS-CoV-2 in the first and second pregnant trimesters were more likely to have children with developmental disorders than those infected in the third trimester of pregnancy. Wu T. et al. (2021) found that SARS-CoV-2 infection in the third pregnant trimester did not increase the risk of developmental disorders at the age of 3 months. We speculate that the dosage and the time window of poly(I:C) exposure can affect the offspring’s neural development and behavior in various degrees. Different strains of animals or people in different states might also respond differently to poly(I:C), LPS or virus. In addition, poly(I:C) from different companies have different properties (Kowash et al., 2019). These problems might be valuable lines of research direction in the future.

### Limitations

This study has some limitations. Firstly, the number of dams was slightly small compared with similar studies (Lins et al., 2018; Gzielo et al., 2021). Secondly, as is described above, different methods constructing the MIA model may produce different results. Poly(I:C) is an analogue of viruses, not a virus. We speculated that MIA induced by poly(I:C) may be inconsistent with MIA induced by virus. A viral infection-induced MIA animal model, such as COVID-19 or cytomegalovirus infection animal model, may be closer to clinical practice. Thirdly, we did not access the serum level of IL-6 in Part Ⅰ. We consider that collecting blood from dams *via* caudal vein 3 h and 6 h after poly(I:C) injection on E 14 may affect the accuracy of basal maternal care and maternal behavior. The causal relationship among MIA, abnormal maternal behavior and autism-like behavior in offspring needs to be explored with more complicated model.

## Conclusion

In general, our present study showed that poly(I:C)-induced MIA dams displayed abnormal static nursing behavior as well as reduced serum levels of testosterone and AVP, accompanied with delayed early development and elevated self-grooming behavior in male offspring. This is the first study systematically evaluating both dams’ maternal behavior and offspring’s autism-like behaviors in one model of MIA. More in-depth and detailed studies exploring the relationship between maternal behavior in dams and autism-like behaviors in offspring as well as the internal mechanisms will be carried out in our future research.

## Data Availability

The original contributions presented in the study are included in the article/supplementary material, further inquiries can be directed to the corresponding author.
